# Ethnic Differences in Facilitators and Barriers to Lifestyle Management After Childbirth: A Multi-Methods Study Using the TDF and COM-B Model

**DOI:** 10.3390/nu17020286

**Published:** 2025-01-14

**Authors:** Mingling Chen, Maureen Makama, Helen Skouteris, Lisa J. Moran, Cheryce L. Harrison, Siew Lim

**Affiliations:** 1 Department of Endocrine and Metabolic Diseases, Shanghai Institute of Endocrine and Metabolic Diseases, Ruijin Hospital, Shanghai Jiao Tong University School of Medicine, Shanghai 200025, China; 2 Shanghai National Clinical Research Center for Metabolic Diseases, Key Laboratory for Endocrine and Metabolic Diseases of the National Health Commission of the PR China, Shanghai Key Laboratory for Endocrine Tumor, Ruijin Hospital, Shanghai Jiao Tong University School of Medicine, Shanghai 200025, China; 3 Monash Centre for Health Research and Implementation, Monash University, 43-51 Kanooka Grove, Clayton, VIC 3168, Australia; 4 Health and Social Care Unit, School of Public Health and Preventive Medicine, Monash University, 553 St. Kilda Road, Melbourne, VIC 3004, Australia; 5 Warwick Business School, University of Warwick, Scarman Road, Coventry CV4 7AL, UK; 6 Diabetes and Vascular Medicine Unit, Monash Health, 246 Clayton Road, Clayton, VIC 3168, Australia; 7 Eastern Health Clinical School, Monash University, 5 Arnold Street, Box Hill, VIC 3128, Australia

**Keywords:** behaviour change, ethnic differences, healthy lifestyle, multi-methods study, postpartum women

## Abstract

**Background**: Understanding ethnic differences in factors influencing healthy lifestyles postpartum is vital for informing effective lifestyle engagement strategies for women from specific ethnic groups. We aimed to explore ethnic differences in facilitators and barriers to lifestyle management among women after childbirth. **Methods**: In this multi-methods study, women within 5 years of childbirth in Australia were recruited in a cross-sectional survey (n = 478) and semi-structured interviews (n = 17). Ethnicity was categorised as Oceanian, Asian and Other, according to the Australian Bureau of Statistics. Chi-square tests were used to compare the survey responses between groups. Qualitative data were thematically analysed, with identified themes mapped to the Theoretical Domains Framework (TDF) and Capability, Opportunity, Motivation and Behaviour (COM-B) model. **Results**: Both Oceanian and Asian women had a range of facilitators and barriers to lifestyle management relating to capability (e.g., knowledge of a healthy lifestyle), opportunity (e.g., time availability) and motivation (e.g., enjoyment in exercise). However, Asian women were more likely to report knowing the importance of a healthy lifestyle (*p* = 0.026), having better practical skills (*p* = 0.004), having a flexible work arrangement (*p* = 0.008) and being able to access a conducive environment (*p* = 0.040) as important factors to maintain a healthy lifestyle, compared with Oceanian women. In addition, Asian women suggested a need to address cultural barriers around parenting and postpartum practices. **Conclusions**: Asian women encountered additional barriers to lifestyle management after childbirth compared with Oceanian women. Future interventions should integrate strategies corresponding to these challenges to improve lifestyle engagement in Asian women.

## 1. Introduction

The prevalence of obesity in women is increasing worldwide, with an expected rise to 21% by 2025 [1]. Postpartum weight retention is a significant contributor to maternal obesity development, and obesity-related comorbidities (e.g., type 2 diabetes) increase the risk of adverse outcomes for both the mother and the offspring in subsequent pregnancies [2]. It is documented that postpartum weight retention varies by ethnicity and is more pronounced in certain ethnic groups [3]. Observational studies in Europe and the US have demonstrated that women from Asian, Middle Eastern and African backgrounds have greater weight retention postpartum compared with those from European origin [4,5,6]. Some ethnic groups, such as Asian, African and Indigenous women, are also at higher risk of developing cardiovascular disease and type 2 diabetes [7,8,9]. Thus, lifestyle intervention including optimization of diet and physical activity in the postpartum period is key to promoting weight loss after pregnancy and reducing long-term health risks among women [3], especially in these priority populations.

However, poor engagement and high attrition have been an issue in postpartum lifestyle interventions [3]. The main barriers to postpartum lifestyle change are unique to the life stage, including childcare needs, time constraints, lack of sleep and inadequate social support [10]. Reaching and engaging women from diverse ethnic backgrounds can be more challenging, possibly due to additional barriers in language, cultural beliefs and social norms [11,12,13]. While existing research has examined barriers and enablers to lifestyle management from the perspective of postpartum women [10], few studies have been conducted focusing on differences in these factors between ethnic groups. In prior qualitative studies describing women’s experiences after gestational diabetes including lifestyle management postpartum, lack of knowledge, time constraints and fragmented health system support after birth were common barriers faced by all women [12,14]. Additional barriers for women from diverse ethnic backgrounds (e.g., South Asian, Chinese) included a lack of culture-specific health advice, postpartum practices, cultural preferences in food and poor support from family and partners [12,14]. Nevertheless, these studies did not apply a theoretical framework to comprehensively understand the influences across attitudinal, skill, behavioural and experiential factors on lifestyle behaviour change and possible ethnic differences on these influences.

Theoretical frameworks can provide a comprehensive understanding of the behaviour change process and therefore inform the design of effective interventions [15]. The Capability, Opportunity, Motivation and Behaviour (COM-B) model proposes that behaviour is a result of the interaction between physical and psychological capabilities, physical and social opportunities and reflective or automatic motivations [16]. The Theoretical Domains Framework (TDF) is an expansion of the COM-B model, comprising 14 domains synthesised from 33 behaviour change theories [17]. Both COM-B and TDF form the hub of the Behaviour Change Wheel, a comprehensive tool to characterise and design interventions [16]. In this study, we used the TDF and COM-B model to identify facilitators and barriers influencing lifestyle behaviour change that can be mapped to specific behavioural strategies to inform intervention development.

In Australia, the largest demographic groups are people born in Oceania (e.g., Australian, New Zealander) followed by those born in Asia (e.g., Indian, Chinese), which constitute the fastest-growing overseas-born populations in recent years [18]. These Asian-born women are more likely to experience postpartum weight retention and obesity-related chronic diseases such as type 2 diabetes compared with Australian-born women [19,20,21]. Thus, intervention efforts are urgently needed to address the health disparities between the two groups. The aim of this study was to examine ethnic differences in facilitators and barriers to lifestyle management across attitudes, skills and behaviours that may be shaped by past experiences among women after childbirth in Australia, with a main focus on Oceanian and Asian women. By using the behavioural frameworks of TDF and COM-B, the ethnic differences in the determinants of lifestyle behaviour change identified in this study will aid the development of effective postpartum lifestyle interventions for different cultures.

## 2. Materials and Methods

### 2.1. Study Design

This study used a multi-methods design involving a quantitative survey and qualitative interviews. The survey provided a broad overview of factors influencing women’s lifestyle management in a larger sample, while the interviews offered an in-depth understanding of women’s perspectives and needs. Eligibility criteria for both methods included women aged 18 years or above, within 5 years of childbirth, living with their youngest child in Australia and not currently pregnant. The survey and interviews were conducted independently and there was no overlap between participants from the two methods. The details of this study have been previously published [22]. This study received ethical approval from the Monash University Human Research Ethics Committee (project number: 29273) and Monash Health Human Research Ethics Committee (reference number: RES-19-0000-685A). Survey participants provided online consent, while interview participants provided audio-recorded verbal consent. The interviews were conducted and reported according to the Consolidated Criteria for Reporting Qualitative Research (COREQ) guidelines [23].

### 2.2. Participants and Data Collection

#### 2.2.1. Survey

Survey participants were recruited online in November 2021 through an external cross-panel market research provider, which has a well-established database of over 350,000 Australian members [24]. To ensure the national representative of the survey participants, the recruitment quota was set by age, sex and location of residence (state/territory) according to the Australian Bureau of Statistics (ABS) [25]. Potential eligible participants were approached by email and screened for eligibility before being directed to the survey. The survey was designed using Qualtrics (Qualtrics, Provo, UT, USA) [26]. The questionnaire was developed by the research team to understand women’s facilitators and barriers to lifestyle management after childbirth, their intervention preferences, health behaviours (including diet, physical activity and sleep patterns), psychological distress, parenting roles and risk perception of type 2 diabetes and cardiovascular disease (Supplementary File S1). The current study focused on ethnic differences in facilitators and barriers to lifestyle management. To detect the differences in facilitators and barriers between ethnic groups at a small effect size of 0.15 based on the analogous literature, a sample size of 429 was required to gain the statistical power of 80% with an α level = 0.05.

The survey comprised 32 statements across the capability, opportunity and motivation domains related to perceived facilitators and barriers to lifestyle management, adapted from the COM-B Self-Evaluation Questionnaire V1 [27]. Participants were asked to select any of the statements in each of the domains that would affect their participation in a lifestyle program after childbirth. To the prefacing question, “What do you think it would take for you to participate in the program?”, an example statement under the *motivation* domain would be “I would have to believe in my ability to do it (e.g., have confidence in my ability to prepare healthy meals)”. Demographic information was collected on age, postpartum age, number of children, ethnicity, country of birth, years lived in Australia, marital status, education, employment and household income. Ethnicity was defined as self-reported ethnic or cultural background and categorised into four broad groups based on the standard classification by the ABS [28]: Oceanian (Australian and New Zealander), Asian (North-East Asian, South-East Asian, Southern and Central Asian), Indigenous (Australian Aboriginal, Torres Strait Islanders, Maori and Pacific Islander) and Other (North-West European, Southern and Eastern European, North African and Middle Eastern, North American, South American, Central American, Caribbean Islander, Central and West African, Southern and East African). Due to Chinese (i.e., Mandarin and Cantonese) being the most commonly spoken language other than English in Australia [29], the questionnaire was provided in English and Chinese to facilitate participation. The survey was pilot-tested in four women (data not included in analysis) and minor amendments were made to improve the clarity of questions and to optimise the survey flow.

#### 2.2.2. Semi-Structured Interviews

To gain an in-depth understanding of the experiences and needs of the two main cultural groups in Australia [18], the semi-structured interviews included women from Oceanian (i.e., Australian and New Zealander) and Asian backgrounds only. The interviews were conducted from May 2020 to July 2020 and in May 2022, both during the WHO-declared COVID-19 pandemic. As the first round of interviews (2020) included a small number of Asian women (n = 4), we conducted a second round of interviews (2022) to include more women from this group. Purposive sampling was employed via researchers’ networks, word of mouth, snowballing technique and the Cardiometabolic Health Implementation Research in Postpartum (CHIRP) women consumer group [30]. All the interviews were carried out on Zoom (Zoom version 5.4.2, Zoom Video Communications Inc., San Jose, CA, USA) to facilitate accessibility for participants. The interview duration varied between 30 and 50 min. In total, 18 eligible participants expressed interest, one of whom was not contactable for the interview. As a result, 17 participants completed the interviews (Oceanian n = 8, Asian n = 9).

The interviews followed a topic guide to explore participants’ facilitators and barriers to lifestyle management after childbirth (Supplementary File S2), which was pilot-tested in two women before the study (data not included in the analysis). Interviews were conducted by a female registered dietitian or doctoral researcher with expertise in lifestyle interventions. Due to the nature of convenience sampling, some participants had a prior relationship with the researchers and were aware of the study context. All the interviews were conducted in English as per participants’ preferences, although English and Chinese languages (i.e., Mandarin and Cantonese) were offered for the participants. Interviews were audio-recorded and transcribed verbatim using a professional transcription service. The transcripts were sent to all participants to check for accuracy and five participants (Oceanian n = 2, Asian n = 3) responded, providing their approval with no changes suggested.

### 2.3. Data Analysis

#### 2.3.1. Survey

Descriptive statistics were used to summarise the demographic characteristics and perceived capability, opportunity and motivation needed for lifestyle management by ethnic groups. Continuous variables were presented in means ± standard deviations and categorical variables in percentages. The statements based on the COM-B constructs were mapped to the TDF domains [17] to assist future intervention functioning and policy making. As a small number of Indigenous women (n = 27) were included in the survey, and they have different cultural barriers and health needs compared with women from other ethnic backgrounds [31], the data pertaining to the Indigenous group are presented separately (Tables S1 and S2). Chi-square tests were performed to investigate the differences in self-evaluation of capability, opportunity and motivation between the Oceanian, Asian and Other ethnic groups. When a significant overall difference was detected, pairwise differences were tested including a Bonferroni correction for multiple comparisons. In this study, while the Other ethnic group was retained for the purpose of a comprehensive analysis, the interpretation focused on Oceanian and Asian women. A two-tailed *p*-value < 0.05 was considered statistically significant for all analyses. Analyses were undertaken using SAS 9.4 (SAS Institute Inc., Cary, NC, USA).

#### 2.3.2. Semi-Structured Interviews

Qualitative data were coded and managed using NVivo 12 (QSR International Pty Ltd., Perth, Australia). One researcher independently coded all the transcripts through an open-coding approach. To ensure reliability, a subsample of transcripts (at least 10%) was coded by a second researcher until consensus coding (at least 90%) was reached between the coders. Thematic analysis was conducted according to the Braun and Clarke method [32]. Subthemes and themes were iteratively derived from the initial codes and subsequently mapped to the COM-B and TDF domains. The themes were developed and refined by two researchers. Deductive mapping was carried out according to the definitions of the COM-B and TDF components [16,17]. Data saturation was achieved within the Oceanian and Asian groups, with no more new themes generated from the transcripts.

#### 2.3.3. Data Integration

Data integration from the survey and interviews was conducted at the reporting and interpretation stage using a weaving approach, which presented both the qualitative and quantitative findings together through the narrative on a theme-by-theme basis under each COM-B and TDF domain [33].

## 3. Results

Of 874 participants approached and screened, 577 were eligible and consented to participate. After excluding data that were missing on the COM-B components (n = 62) or ethnicity (n = 10) or were from the Indigenous group (n = 27), 478 participants were included in the analysis (Oceanian n = 239, Asian n = 174 and Other n = 65). The majority of participants (99.4%) completed the questionnaire in English.

The semi-structured interviews included 17 participants (Oceanian n = 8, Asian n = 9). The demographic characteristics of survey and interview participants are presented in Table 1 and Table S3. Table 2 shows the survey responses, and Table 3 shows the themes identified from the interviews. Both quantitative and qualitative results are organised according to the COM-B and TDF domains. A full list of subthemes and codes derived from the interviews is provided in Table S4.

### 3.1. COM-B: Psychological Capability; TDF: Knowledge, Skills, Behavioural Regulation

#### 3.1.1. Having and Obtaining Knowledge of Healthy Lifestyle

The interviews identified adequate knowledge as a facilitator of lifestyle management for both Oceanian and Asian women. Both ethnic groups stated that knowing the benefits of a healthy lifestyle or the health consequences of unhealthy habits would keep them motivated in sustainable behaviour change. On the other hand, the lack of ability to find evidence-based information on healthy lifestyles or information that met their health needs was considered a barrier to making appropriate behaviour changes. Although the survey similarly showed a need to know the importance of a healthy lifestyle (e.g., have a better understanding of how foods affect my health) to engage in lifestyle management for all ethnic groups, this was significantly more pronounced among Asian women than in Oceanian women (68.4% vs. 55.7%, *p* = 0.026). Moreover, Asian women were more likely to report a need for having better physical skills (e.g., learning how to cook healthy meals for the family) as compared with Oceanian women (51.7% vs. 36.0%, *p* = 0.004).

#### 3.1.2. Prioritising, Organising and Planning for Healthy Lifestyle

In the interviews, both Oceanian and Asian women thought the ability to prioritise self-care facilitated their lifestyle management, which findings were supported by over half of the survey participants from these groups. Prioritising self-care could be achieved by organizing and planning a healthy lifestyle ahead to address time constraints and commitments to multiple demands in the postpartum period. Incorporating a healthy lifestyle into daily activity was another strategy, such as doing exercise while walking the dog or during the baby’s nap time.

### 3.2. COM-B: Physical Capability; TDF: Skills

#### Fatigue, Lack of Sleep and Mental Health Challenges

In the interviews, both Oceanian and Asian women reported that lack of sleep was the biggest barrier in the early postpartum period due to the care of the baby. Sleep deprivation led to fatigue, low energy levels and less motivation to manage health, resulting in an unhealthy diet (e.g., increased sugar or caffeine intake) and insufficient physical activity. Also, the multiple demands of motherhood contributed to adverse mental health such as anxiety, which exacerbated poor sleep and unhealthy eating behaviours. In contrast, women who had no sleep problems or were able to address mental health issues felt they were ready to engage in lifestyle management. Similarly, in the survey, over half of women in each ethnic group agreed that they would participate in a healthy lifestyle if they had more physical strength (e.g., having the fitness to exercise) or had the ability to overcome physical limitations (e.g., recovery from childbirth; lack of sleep) and mental obstacles (e.g., stress or negative thoughts about self).

### 3.3. COM-B: Physical Opportunity; TDF: Environmental Context and Resources

#### 3.3.1. Limited Time Availability with Competing Priorities

The survey revealed that, for all ethnic groups, having more time to manage their lifestyle (e.g., creating a specific time during the day to exercise) was the most needed physical opportunity. This was further illustrated by the interview discussions where both Oceanian and Asian women reported time constraints as a major challenge. Multiple commitments including childcare needs, motherly duties, household chores and work led to a busy schedule in women’s daily life. These commitments were often prioritised over women’s own health, limiting their ability to participate in exercise, select healthy foods and prepare healthy meals. For working mothers, a flexible work arrangement was thought as a facilitator to their lifestyle management. Some women found working from home during the COVID-19 lockdown was beneficial, as this format enabled the balance between work, childcare and their self-care. Asian women were more likely to need a flexible work arrangement (e.g., part-time employment) compared with Oceanian women (54.6% vs. 39.8%, *p* = 0.008), as indicated in the survey.

#### 3.3.2. Physical Access to Healthy Lifestyle Resources

In the interviews, both Oceanian and Asian women thought access to practical resources, such as community facilities (e.g., park, gym, swimming pool), home-based exercise equipment, online exercise programs and food services (e.g., food delivery service, healthy or affordable food availability), facilitated their lifestyle management. For example, food delivery service in the living area allowed them to select healthy foods online, enabling time to meet other family responsibilities and self-care demands. Conversely, other environmental contexts, including unsafe living neighbourhoods, poor weather conditions as well as the COVID-19 lockdown, were cited as barriers due to the limited opportunities for outdoor activities. The survey showed significant group differences in the need for these environmental resources. Asian women were more likely to report a need for access to a conducive environment for a healthy lifestyle (e.g., access to recreational facilities and parks) compared with Oceanian women (43.7% vs. 31.8%, *p* = 0.040), while Oceanian women were more likely to report a need for necessary materials (e.g., exercise equipment) to participate in a healthy lifestyle relative to the Other ethnic group (45.2% vs. 26.2%, *p* = 0.017). In addition, both Oceanian and Asian women in the interviews expressed that financial constraint was a barrier to lifestyle management, limiting their ability to afford healthy foods, exercise equipment, gym membership or fitness classes. The constrained finance mainly derived from reduced salary on maternity leave and increased household expenditure after having a child.

### 3.4. COM-B: Social Opportunity; TDF: Social Influences

#### 3.4.1. Practical Support on Childcare and Household Chores

Across both the survey and interviews, having practical support from partners and family played an important role in maintaining a healthy lifestyle for each ethnic group. Both Oceanian and Asian women stated that supportive partners and family freed them from childcare and household chores, making time for them to rest, exercise and socialise with friends. However, some participants reported lacking such kind of support due to having a busy partner or no family around after relocation or migration. Childcare services such as centre-based daycare and babysitting were considered as another important source of practical support, especially when no assistance was available from family members.

#### 3.4.2. Mental and Wellbeing Support Especially for Migrants

The interviews additionally identified the importance of social interaction and emotional support from family, friends and community for both Oceanian and Asian women, which helped them navigate through the challenging times during the transition into motherhood. This was especially important in Asian women who frequently reported loneliness following childbirth due to the lack of social connection as a migrant in the new country. On the other hand, women uncovered that having a partner sharing the same health values could greatly facilitate their engaging in healthy lifestyles and maintaining wellbeing, although this statement was only supported by less than 50% of the survey participants in each ethnic group.

#### 3.4.3. Social Norms Around Parenting and Postpartum Practices in Asian Cultures

Social norms were identified in the interviews as a barrier to lifestyle management for Asian women. In Asian cultures, mothers are believed to take the primary responsibility of caring for and nurturing their children. The expected gender roles often created pressure on mothers and limited their abilities for self-care. Asian women also expressed concerns about the traditional postpartum practices, in which they were advised to remain indoors with limited activities. They perceived that the restrictions during the postpartum confinement period (30–40 days following childbirth) could impede their early engagement in lifestyle management.

### 3.5. COM-B: Reflective Motivation; TDF: Intentions, Beliefs About Consequences and Capabilities, Goals, Social/Professional Role and Identity

#### Difficulties with Prioritising Self and Maintaining Motivation

In the interviews, both Oceanian and Asian women found it difficult to prioritise themselves and maintain motivation in changing lifestyle behaviours. In their perceptions, children and the family were in the first place, and their own needs came last on the priority list. Some participants had feelings of guilt when taking time out for themselves and leaving their children behind. Thus, being self-motivated was an important facilitator to making behaviour change. This could be achieved through a number of ways including self-talk, setting a goal and tracking the progress. The survey additionally showed that women would engage in lifestyle management if they felt that they needed to do it enough (e.g., believe that my own health is important), believed that it would be a good thing to do (e.g., it will help me cope emotionally or make me feel better), or believed that it was good for their children (e.g., I am being a good example for my child). Oceanian women were more likely to report they felt the need to do it enough when compared with the Other ethnic group (66.1% vs. 47.7%, *p* = 0.020). For Asian women, a slightly higher proportion of them reported it would have to fit their cultural and/or religious beliefs (e.g., beliefs about the type of food to eat when breastfeeding), as compared to Oceanian women (27.6% vs. 12.1%, *p* < 0.001).

### 3.6. COM-B: Automatic Motivation; TDF: Emotion

#### Enjoyment in Exercise or Eating Behaviours

From the survey, the majority of participants (over 60%) in each ethnic group reported that they would participate in a healthy lifestyle if they wanted to do it enough (e.g., enjoy eating healthy or exercising) or developed a habit of doing it (e.g., get into a pattern of eating healthy without having to think). Similarly, in the interviews, identifying exercise as an emotional coping strategy was a facilitator to being physically active for both Oceanian and Asian women. Women found exercising alleviated emotional strain, created mental space and thus made them energetic in routine life. Enjoyment in cooking was recognised as a facilitator to healthy eating whereas snacking, sweet cravings and preferring taste-dense foods served as barriers.

## 4. Discussion

This study explored facilitators and barriers to lifestyle management after childbirth in women from different ethnic backgrounds using the TDF and COM-B model, with a focus on the Oceanian and Asian groups in Australia. The survey and interviews identified a range of facilitators and barriers influencing capability, opportunity and motivation for lifestyle management in both Oceanian and Asian women, such as limited time availability, competing priorities and enjoyment in exercise or eating behaviours, as similarly found in existing literature [10]. However, ethnic differences were present across the COM-B and TDF domains. Specifically, Asian women were more likely to report a need for knowing the importance of a healthy lifestyle, having better physical skills, having a flexible work arrangement and being able to access a conducive environment to participate in a healthy lifestyle compared with Oceanian women. Asian women also suggested a need to address cultural barriers in traditional parenting and postpartum practices.

In terms of *capability*, ethnic differences were revealed in the *knowledge* and *skills* domains. Our results emphasised the need for knowledge on the importance of a healthy lifestyle as well as better physical skills (i.e., the ability to acquire health behaviours through practice) in Asian women. Previous studies have found that one of the main barriers to adopting a healthy lifestyle in postpartum women is limited knowledge of the relationship between lifestyle behaviours and disease prevention [10]. This seems to be more pronounced in Asian migrant women, as observed here, which may be partially explained by their lower access to or unfamiliarity with health services in the new country due to culturally inappropriate services or language barriers [34]. It is imperative to provide culturally appropriate education for women from diverse ethnic backgrounds to address this need, particularly in describing the link between health behaviours and comorbidities of obesity (e.g., type 2 diabetes). To further improve behaviour change, the provision of knowledge should also be supplemented with practical skills such as how to prepare healthy meals. According to the social cognitive theory, individuals must know what to do and how to do it in order to successfully perform a behaviour [35]. The literature shows that interventions involving cooking skill development can improve confidence, satisfaction and enjoyment in cooking, which are associated with improved eating behaviours and weight-related outcomes [36].

With regard to *opportunity*, ethnic differences were evident in both the domains of *environmental context and resources* and *social influences*. Our results showed that the perceived need for an appropriate environment for healthy eating and exercise was more pronounced in Asian women compared with Oceanian women. This may be because new migrants, which accounted for almost 30% of the Asian participants in our study (≤5 years of residence), tend to live in shared or rental housing that may limit their space or privacy for certain home-based activities [37]. Moreover, physical distance, lack of transportation and safety concerns due to perceived crime rates have been frequently reported as barriers to physical activity outside of home (e.g., walking, exercise classes) among Asian groups in Australia [38,39]. The lack of culturally appropriate settings for physical activity could also be a barrier, as women in some Asian subgroups are not allowed or feel uncomfortable using public exercise facilities with males due to conservative gender norms in their cultures and religions [40]. Thus, there is a need to build a more accessible and supportive environment for maintaining a healthy lifestyle in Asian communities. Furthermore, Asian women in our study were more likely to indicate a need for having a flexible work arrangement to facilitate their lifestyle management. This may be explained by the nature of occupation (e.g., frontline care that migrants are often employed in) [41] or potentially longer work hours in the Asian participants, one-third of which were full-time employed. The Household, Income and Labour Dynamics in Australia Survey showed that although all women have reduced work hours following childbirth, migrant women experience a smaller reduction in work hours than native-born women [42]. The relatively longer work time after childbirth in migrant women is possibly due to their work-family preferences or greater financial necessity [42], suggesting potentially reduced capacity to manage lifestyle among migrant mothers, especially in the absence of adequate childcare support. Previous studies indicated that women working full-time have greater weight gain and less weight loss compared with those part-time workers [43]. To improve the health of women from ethnically diverse backgrounds, policies and strategies that support healthy lifestyles in working mothers, such as flexible work scheduling and health and wellbeing support in workplace settings, are critical.

Social norms were another important factor influencing women’s opportunities for lifestyle management. We identified parenting norms and traditional postpartum practices as additional barriers to adopting a healthy lifestyle in Asian women as per previous studies [11,12]. To fulfil the culturally expected roles of mothers, Asian women usually perform most of the childcare duties [44] and prioritise their children’s needs and family obligations over the pursuit of personal health. Given the challenges that women are faced with during the transition into parenthood, their abilities to maintain a healthy lifestyle can largely depend on partner support in child-rearing activities [45]. This could be prominent when migrant women have little family support or low accessibility to childcare services in their new countries. Research has shown the positive associations of partner support with maternal mental health and lifestyle behaviours [46], leading to improvement in maternal postpartum anthropometric outcomes even in the absence of specific lifestyle advice [47]. It is therefore crucial to involve partners in supporting postpartum women’s healthy lifestyles, especially among Asian groups. Traditional postpartum practices are common in Asian cultures, which are believed to have health benefits for the mother and the baby through exercise restrictions, dietary regulations, such as increasing calorie intake and avoiding “cold” foods like fruits and vegetables, and other practices including bathing abstinence [48]. The confinement period, which usually lasts one to three months [48], may therefore preclude women from engaging in a healthy lifestyle in the early postpartum stage. Our previous findings have shown that Asian women tend to prefer lifestyle interventions that are initiated later following birth compared with Oceanian women [22]. These ethnic-specific needs should be taken into account when developing and providing culturally appropriate postpartum care in Asian groups.

For *motivation*, we found ethnic differences in the *social/professional role and identity* domain. Compared with Oceanian women, Asian women were more likely to report the need for a healthy lifestyle to fit their cultural or religious beliefs. Previous research has similarly suggested the need to address cultural beliefs (e.g., traditional postpartum practices) in postpartum health behaviours in Asian women [11,48]. However, we noted that this demand was generally low across all ethnic groups compared with other factors mentioned above, suggesting other factors may play a more important role in influencing behaviour change. From the current study, emotion, such as enjoyment in healthy eating and exercise, was the strongest motivating factor for behaviour change in all ethnic groups. Strategies that stimulate enjoyment (e.g., listening to music) [49] and enhance motivation (e.g., motivational interviewing) [50] should be used to support healthy lifestyle engagement among women from all ethnic groups.

Our study has several strengths. To our knowledge, this is the first multi-methods study that examined ethnic differences in facilitators and barriers to lifestyle management in women after childbirth using the TDF and COM-B frameworks. The inclusion of both quantitative and qualitative components allowed data integration from different sources to provide a comprehensive understanding of women’s perspectives and needs across cultures [51]. In addition, by mapping the inductive themes to the TDF and COM-B model, the differential facilitators and barriers identified between ethnic groups can be linked to the intervention functions and serve as key determinants to developing effective interventions for different ethnic groups [16,17].

However, some limitations should be considered. First, the limited number of participants in this study restricted the further breakdown into more precise ethnic groups. Instead, we used a broad categorisation of ethnic groups (i.e., Oceanian, Asian, Indigenous and Other) to allow for comparisons, but at the cost of masking the diversity of cultures within each group. The small sample size of the Indigenous group meant that identifying the differences between this underserved group and other ethnic groups was not possible. Second, some socio-demographic factors were not collected (e.g., working hours, occupation, living neighbourhood), which could have elucidated the findings further. Third, the interview participants in the Asian group were highly educated, fluent in English, and had lived in Australia for over 6 years. Hence, these results may not reflect the experiences and needs of non-English-speaking new migrants. Furthermore, the study results were not stratified by time since childbirth as constrained by the sample size. The potential differences in factors influencing healthy lifestyle behaviours in the earlier and later postpartum periods by ethnicity could be further investigated in future research.

## 5. Conclusions

In comparison with Oceanian women, Asian women were more likely to perceive knowledge and practical skills, a conducive physical environment and flexible work arrangements as important factors in enabling a healthy lifestyle. These may reflect unmet needs in these areas. Opportunities for a healthy lifestyle may be further limited by the prioritisation of cultural parenting and postpartum practices. Given these multilevel barriers unique to Asian women, future interventions should integrate strategies such as providing culturally appropriate dietary and physical activity advice that is compatible with cultural parenting and postpartum practices, information on flexible work scheduling and navigation of environmental and physical resources available to support a healthy lifestyle. Addressing these barriers is key to improving lifestyle engagement, thereby reducing health inequities in postpartum weight retention and chronic disease risks in Asian women.

## Figures and Tables

**Table 1:** Demographic characteristics of survey and interview participants.

Characteristics	Survey			Interview	
	Oceanian (n = 239)	Asian (n = 174)	Other (n = 65)	Oceanian (n = 8)	Asian (n = 9)
Age (years)	33.1 ± 5.8	34.3 ± 4.6	35.1 ± 5.2	36.4 ± 4.7	37.7 ± 4.8
Postpartum age (years)	2.1 ± 1.6	2.5 ± 1.6	2.7 ± 1.7	1.4 ± 0.3	2.1 ± 0.9
Number of children living in the household					
1	76 (31.8)	78 (44.8)	23 (35.4)	3 (37.5)	3 (33.3)
2	102 (42.7)	74 (42.5)	23 (35.4)	3 (37.5)	4 (44.4)
≥3	61 (25.5)	22 (12.6)	19 (29.2)	2 (25.0)	2 (22.2)
Born in Australia					
No	17 (7.1)	156 (89.7)	51 (78.5)	1 (12.5)	9 (100.0)
Years lived in Australia					
≤5 years	1 (0.4)	49 (28.2)	15 (23.1)	0 (0.0)	0 (0.0)
6 to 10 years	8 (3.4)	64 (36.8)	11 (16.9)	1 (12.5)	3 (33.3)
≥11 years	230 (96.2)	61 (35.1)	39 (60.0)	7 (87.5)	6 (66.7)
Marital status					
Never married	19 (8.0)	2 (1.2)	4 (6.2)	1 (12.5)	0 (0.0)
Married	136 (56.9)	161 (92.5)	44 (67.7)	5 (62.5)	9 (100.0)
De facto	67 (28.0)	7 (4.0)	12 (18.5)	1 (12.5)	0 (0.0)
Separated or divorced	16 (6.7)	2 (1.2)	5 (7.7)	1 (12.5)	0 (0.0)
Education					
High school	83 (34.7)	17 (9.8)	10 (15.4)	1 (12.5)	0 (0.0)
Diploma	59 (24.7)	21 (12.1)	15 (23.1)	0 (0.0)	0 (0.0)
Bachelor	55 (23.0)	70 (40.2)	24 (36.9)	1 (12.5)	2 (22.2)
Postgraduate	41 (17.2)	65 (37.4)	16 (24.6)	6 (75.0)	7 (77.8)
Employment					
Full time	72 (30.1)	58 (33.3)	16 (24.6)	4 (50.0)	3 (33.3)
Part-time	92 (38.5)	60 (34.5)	28 (43.1)	4 (50.0)	5 (55.6)
Unemployed	72 (30.1)	54 (31.0)	20 (30.8)	0 (0.0)	1 (11.1)
Household income per year					
≤$49,999	39 (16.3)	19 (10.9)	10 (15.4)	0 (0.0)	1 (11.1)
$50,000 to $99,999	60 (25.1)	62 (35.6)	19 (29.2)	0 (0.0)	0 (0.0)
≥$100,000	129 (54.0)	79 (45.4)	33 (50.8)	6 (75.0)	5 (55.6)
Prefer not to answer	11 (4.6)	14 (8.1)	3 (4.6)	2 (25.0)	3 (33.3)

Data are presented as mean ± standard deviation or n (%). Oceanian: Australian and New Zealander; Asian: North-East Asian, South-East Asian, Southern and Central Asian; Other: North-West European, Southern and Eastern European, North African and Middle Eastern, North American, South American, Central American, Caribbean Islander, Central and West African, Southern and East African.

**Table 2:** Survey responses on capability, opportunity and motivation for participation in lifestyle management after childbirth according to the COM-B and TDF domains.

COM-B Constructs	TDF Domains	Questionnaire Statement: *I would have to…*	Oceanian (n = 239)	Asian (n = 174)	Other (n = 65)	*p*-Value
Psychological capability	Knowledge	Know more about why it was important (e.g., have a better understanding of how foods affect my health)	133 (55.7)	119 (68.4)	35 (53.9)	0.018 ^a^
		Know more about how to do it (e.g., have a better understanding of effective ways to increase exercise)	150 (62.8)	104 (59.8)	34 (52.3)	0.308
	Skills	Know where to find information	147 (61.5)	101 (58.1)	36 (55.4)	0.605
		Know how to create restful time or space for myself	154 (64.4)	99 (56.9)	37 (56.9)	0.242
		Have better physical skills (e.g., learn how to cook healthy meals for the family)	86 (36.0)	90 (51.7)	29 (44.6)	0.006 ^b^
	Behavioural regulation	Know how to organise, plan and prioritise (e.g., exercise during child’s nap time; incorporate into usual routine such as taking the baby for a walk)	130 (54.4)	109 (62.6)	32 (49.2)	0.106
		Have more mental strength (e.g., learn how to resist cravings more)	158 (66.1)	103 (59.2)	44 (67.7)	0.276
		Have more mental stamina (e.g., be able to stick to a plan to eat healthy)	126 (52.7)	80 (46.0)	37 (56.9)	0.229
Physical capability	Skills	Have more physical strength (e.g., having the fitness to exercise)	143 (59.8)	107 (61.5)	34 (52.3)	0.429
		Have more physical stamina (e.g., be able to exercise for longer)	107 (44.8)	80 (46.0)	28 (43.1)	0.919
		Overcome physical limitations (e.g., recovery from childbirth; coping with lack of sleep)	143 (59.8)	102 (58.6)	43 (66.2)	0.561
		Overcome mental obstacles (e.g., managing stress or negative thoughts about self)	144 (60.3)	90 (51.7)	44 (67.7)	0.055
Physical opportunity	Environmental context and resources	Have more time to do it (e.g., create a specific time during the day to exercise)	177 (74.1)	120 (69.0)	48 (73.9)	0.495
		Have a flexible work arrangement (e.g., part-time employment)	95 (39.8)	95 (54.6)	25 (38.5)	0.006 ^c^
		Have enough money to do it (e.g., earn enough to pay for gym membership)	148 (61.9)	99 (56.9)	31 (47.7)	0.109
		Have the necessary materials (e.g., exercise equipment)	108 (45.2)	73 (42.0)	17 (26.2)	0.022 ^d^
		Have it more easily accessible (e.g., online access to the intervention)	116 (48.5)	87 (50.0)	29 (44.6)	0.760
		Have it incorporated with my baby’s appointment (e.g., maternal and child health visits)	102 (42.7)	87 (50.0)	34 (52.3)	0.209
		Have a conducive environment to do it (e.g., access to recreational facilities and parks)	76 (31.8)	76 (43.7)	31 (47.7)	0.012 ^e^
Social opportunity	Social influences	Have more people around me doing it (e.g., be part of a ’crowd’ who are doing it)	95 (39.8)	67 (38.5)	24 (36.9)	0.909
		Have more triggers to prompt me (e.g., have more reminders to exercise at specific times)	87 (36.4)	65 (37.4)	18 (27.7)	0.354
		Have the support of my partner on health issues (e.g., verbal and emotional encouragement)	87 (36.4)	80 (46.0)	29 (44.6)	0.121
		Have practical support from others (e.g., help with childcare and chores from partner, family and friends)	138 (57.7)	84 (48.3)	36 (55.4)	0.158
		Have someone to hold me accountable	68 (28.5)	34 (19.5)	16 (24.6)	0.116
Reflective motivation	Intentions	Feel that I need to do it enough (e.g., believe that my own health is important; feel the need to prioritise self-care)	158 (66.1)	108 (62.1)	31 (47.7)	0.025 ^f^
	Beliefs about consequences	Believe that it would be a good thing to do (e.g., it will help me cope emotionally or make me feel better)	149 (62.3)	107 (61.5)	35 (53.9)	0.451
		Believe that it is good for my children (e.g., I am being a good example for my child)	156 (65.3)	111 (63.8)	44 (67.7)	0.850
	Beliefs about capabilities	Believe in my ability to do it (e.g., have confidence in my ability to prepare healthy meals)	119 (49.8)	86 (49.4)	31 (47.7)	0.956
	Goals	Develop better plans for doing it (e.g., have a clearer and better-developed plan for eating healthy)	129 (54.0)	81 (46.6)	31 (47.7)	0.295
	Social/professional role and identity	It would have to fit my cultural and/or religious beliefs (e.g., beliefs about the type of food to eat when breastfeeding)	29 (12.1)	48 (27.6)	11 (16.9)	<0.001 ^g^
Automatic motivation	Emotion	Feel that I want to do it enough (e.g., enjoy eating healthy or exercise)	168 (70.3)	116 (66.7)	44 (67.7)	0.724
		Develop a habit of doing it (e.g., get into a pattern of eating healthy without having to think)	162 (67.8)	113 (64.9)	40 (61.5)	0.607

COM-B, Capability, Opportunity, Motivation and Behaviour; TDF, Theoretical Domains Framework. Data are presented as n (%). Oceanian: Australian and New Zealander; Asian: North-East Asian, South-East Asian, Southern and Central Asian; Other: North-West European, Southern and Eastern European, North African and Middle Eastern, North American, South American, Central American, Caribbean Islander, Central and West African, Southern and East African. ^a^ Significant pairwise difference between Oceanian and Asian (Bonferroni-adjusted *p* = 0.026). ^b^ Significant pairwise differences between Oceanian and Asian (Bonferroni-adjusted *p* = 0.004). ^c^ Significant pairwise differences between Oceanian and Asian (Bonferroni-adjusted *p* = 0.008). ^d^ Significant pairwise differences between Oceanian and Other (Bonferroni-adjusted *p* = 0.017). ^e^ Significant pairwise differences between Oceanian and Asian (Bonferroni-adjusted *p* = 0.040). ^f^ Significant pairwise differences between Oceanian and Other (Bonferroni-adjusted *p* = 0.020). ^g^ Significant pairwise differences between Oceanian and Asian (Bonferroni-adjusted *p* < 0.001).

**Table 3:** Interview participants’ perspectives on facilitators and barriers to lifestyle management after childbirth according to the COM-B and TDF domains.

COM-B Constructs	TDF Domains	Themes	Ethnic Groups	Representative Quotes
Psychological capability	Knowledge, skills	Having and obtaining knowledge of healthy lifestyle	Both	*“There’s probably not a lot of information that I wouldn’t know where to find if I needed it.”—*O8 (New Zealander) *“I think with my current health knowledge, I’m more motivated and I know what are the consequences if I don’t manage my health properly.”*—A8 (Southern Asian)
	Behavioural regulation	Prioritising, organising and planning for healthy lifestyle	Both	*“I have one afternoon a week I have help from a babysitter, so that I can exercise. So making the time and prioritizing exercise helps.”*—O4 (Australian) *“Basically I do about 30 min exercise every day and it’s mostly just about weight and power… It’s only about half an hour. So you can fit into that nap time perfectly.”*—A3 (North-East Asian)
Physical capability	Skills	Fatigue, lack of sleep and mental health challenges	Both	*“I guess the biggest challenge for me at the moment is probably not getting enough sleep. I think if I don’t have enough sleep, then I’ve kind of got less energy and motivation to do all of the other things that are important.”*—O1 (Australian) *“When you are anxious about something, then you just get fixated on a problem and then you forget about being healthy or having a balancing, those things that are important to you in life.”*—A4 (North-East Asian)
Physical opportunity	Environmental context and resources	Limited time availability with competing priorities	Both	*“If it’s a particularly busy week or there’s a child home from daycare, and you’re trying to prioritize getting your work done, exercise will certainly be the thing that slips or get pushed to the bottom of the priority list.”*—O2 (Australian) *“I think it’s just the busyness of life. Like, keeping with online helping and doing the house chores, and attending to the need of this little one. So sometimes you kind of forget, ‘Oh, what time is it now?’”*—A1 (South-East Asian)
		Physical access to healthy lifestyle resources	Both	*“A gym membership would be hugely expensive. On the forums, I can see women saying, I can’t afford this month. I can’t afford that month. I haven’t been able to… So exercise comes with a cost, even if it’s through equipment or memberships.”*—O5 (Australian) *“It’s not a safe neighborhood at all. I don’t feel safe. That’s probably the main reason I don’t do my night anymore. That evening walk from 7:30 to 8:30.”*—A9 (Southern Asian)
Social opportunity	Social influences	Practical support on childcare and household chores	Both	*“Unfortunately given the kids and working and multiple commitments and having a husband that’s not home very often… I’m not a solo parent, but I do every pick up, every drop off, and every meal preparation, so I don’t have a lot of time to exercise.”—*O4 (Australian) *“For myself I think maybe it’s the help from the husband. Because when I do these, he needs to look after the kids. So it’s the husband’s cooperation and understanding.”*—A2 (North-East Asian)
		Mental and wellbeing support especially for migrants	Both	*“I definitely have a supportive partner who shares the same goals and we both want to be in the best health that we can be for ourselves and for our kids. We support each other to take some time to exercise.”*—O2 (Australian) *“I feel having kids in Australia is a very lonely process for the first year if you don’t have anybody helping you… It’s very lonely, when I can go out and start having some adult conversation, I will be in tears already.”—*A2 (North-East Asian)
		Social norms around parenting and postpartum practices in Asian cultures	Asian	*“When you are a mother, it is your duty to do everything according to our family. Because we are raised in Asian culture, and they believe it’s a mother’s responsibility to take care of the child.”*—A5 (Southern Asian) *“We have different traditions and customs and stuff like that. So you have to go through that one and a half month of postpartum thing. They’re more important than your health… Once you have given birth, you just need to stay in the bed, most of the time cover yourself, cover the baby, cover yourself, cover the baby. Don’t go anywhere.”*—A5 (Southern Asian)
Reflective motivation	Intentions, goals	Difficulties with prioritising self and maintaining motivation	Both	*“At the early time when my first daughter was born, I definitely didn’t prioritize my own wellbeing and exercise or physical activity very much at all.”*—O8 (New Zealander) *“I do feel less motivated to do it because sometimes even if these only take about 30 min. You’ve got to push yourself hard sometimes to keep doing it.”*—A3 (North-East Asian)
Automatic motivation	Emotion	Enjoyment in exercise or eating behaviours	Both	*“Being a dietitian I’m quite a good cook. So that definitely helps. Because I can make healthy food pretty easily without any issues. For me, cooking is actually a way to switch off, and tune out. So that’s kind of my relaxation time.”*—O4 (Australian) *“Just like any Indian, I am fond of the taste, for example, more important than a healthy diet.”*—A6 (Southern Asian)

COM-B, Capability, Opportunity, Motivation and Behaviour; TDF, Theoretical Domains Framework.

## Data Availability

The data used in this study are available from the corresponding author upon reasonable request.

## Supplementary Materials

The following supporting information can be downloaded at: https://www.mdpi.com/article/10.3390/nu17020286/s1, Supplementary File S1: Online survey; Supplementary File S2: Interview guide; Table S1: Demographic characteristics of Indigenous participants in the survey; Table S2: Survey responses on capability, opportunity and motivation for participation in lifestyle management after childbirth according to the COM-B and TDF domains in Indigenous participants; Table S3: Location of residence and ethnicity of survey and interview participants; Table S4: Codes, subthemes and themes on women’s perspectives derived from interviews.
