# Supplementary material for: Ethnic Differences in Facilitators and Barriers to Lifestyle Management After Childbirth: A Multi-Methods Study Using the TDF and COM-B Model
Source: Nutrients. 2025 Jan 14;17(2):286. doi: 10.3390/nu17020286 (PMC11769254; doi:10.3390/nu17020286)
Supplement: Supplementary file 1 [file nutrients-17-00286-s001.zip › Supplementary File S1.pdf]

English (United Kingdom)

Screening questions

Before you begin, please answer these questions to confirm your eligibility for the survey.

Do you identify as female?

- ◯ Yes
- ◯ No

Are you an Australian citizen or permanent resident?

- ◯ Yes
- ◯ No

Are you 18 years old or above?

- ◯ Yes
- ◯ No

Have you given birth to a baby in the last 5 years and currently live with the child?

- ◯ Yes
- ◯ No

Are you currently pregnant?

- Yes
- No

How old is your youngest child?

- Less than 6 months old
- 6 months to less than 1 year old
- 1 year old
- 2 years old
- 3 years old
- 4 years old
- 5 years old

Demographics

Background

The following questions are about your background. Please answer as accurately as possible.

What state or territory are you from?

- Victoria
- Queensland
- New South Wales
- Tasmania
- Northern Territory
- Western Australia
- Australian Capital Territory
- South Australia

What is your cultural or ethnic background?

- ○ Australian (excluding Indigenous Australian)
- ○ New Zealander (excluding Pacific Islander)
- ○ Australian Aboriginal, Australian South Sea Islander, Torres Strait Islander, Maori, Melanesian and Papuan, Micronesian, Polynesian
- ○ North-West European (British, Irish, Western European, Northern European)
- ○ Southern and Eastern European (Southern European, South Eastern European, Eastern European)
- ○ North African and Middle Eastern (Arab, Jewish, Peoples of the Sudan, other North African and Middle Eastern)
- ○ South-East Asian (e.g. Filipino, Vietnamese, Malay, Indonesian, Thai)
- ○ North-East Asian (e.g. Chinese, Korean, Japanese)
- ○ Southern and Central Asian (e.g. Indian, Sri Lankan, Nepalese, Pakistani)
- ○ North American (e.g. American, Canadian)
- ○ South American (e.g. Brazilian, Colombian, Chilean, Argentinian)
- ○ Central American (e.g. Mexican)
- ○ Caribbean Islander (e.g. Cuban)
- ○ Central and West African (e.g. Nigerian)
- ○ Southern and East African (e.g. South African, Zimbabwean, Kenyan, Ethiopian)
- ○ I prefer not to say
- ○ Other, please specify ____________

Do you self-identify as a migrant with a Chinese background or someone from a Chinese migrant family?

- ○ Yes
- ○ No

Does your partner self-identify as a migrant with a Chinese background or someone from a Chinese migrant family?

- ○ Yes
- ○ No

What is your postcode?

What is your age in years?

Were you born in Australia?

- ○ Yes
- ○ No

If not born in Australia, how long ago did you migrate to Australia?

- ○ 5 years or less
- ○ 6 - 10 years
- ○ 11 - 15 years
- ○ 16 years or more

What is the highest level of education you have completed?

- ○ Primary/elementary school or less
- ○ Secondary/high school
- ○ Diploma
- ○ Advanced diploma
- ○ University degree
- ○ Graduate/postgraduate degree
- ○ I have never been to school
- ○ I don't know
- ○ I prefer not to answer

How many adults (18 years and above) live with you (i.e. excluding yourself) at home currently?

[▾]

How many children (less than 18 years) live with you at home currently?

[▾]

What are the ages of the children in **years**? (If less than 1 year old, please specify in months)

Child 1	
Child 2	
Child 3	
Child 4	
Child 5	
Child 6	
Child 7	
Child 8	
Child 9	
Child 10	
Child 11	
Child 12	

What is your current marital status?

- ○ Never married
- ○ Married (opposite sex)
- ○ Married (same sex)
- ○ De facto (opposite sex)
- ○ De facto (same sex)
- ○ Separated
- ○ Divorced
- ○ Widowed
- ○ I prefer not to say

How would you describe your current employment status?

- Homemaker
- Full-time employment
- Part-time employment
- Casual employment
- Student
- Retired
- Government assistance
- Government disability support
- I don’t know
- I prefer not to answer

What is your annual household income before tax?

- $0-$24,999
- $25,000-$49,999
- $50,000-$74,999
- $75,000-$99,999
- $100,000-$124,999
- $125,000-$149,999
- >$150,000
- I don't know
- I prefer not to answer

Have you ever had, been diagnosed, or treated for (tick all that apply):

- ☐ Diabetes (excluding gestational diabetes)
- ☐ Gestational diabetes (diabetes onset in pregnancy)
- ☐ Gestational hypertension (high blood pressure that started during pregnancy)
- ☐ Pre-eclampsia
- ☐ Pre-term birth delivery (delivery of a baby before 37 weeks of pregnancy)
- ☐ Delivery of a baby with birth weight less than 2500g after 37 weeks of pregnancy
- ☐ Polycystic ovary syndrome
- ☐ Infertility
- ☐ Menopause
- ☐ None of the above
- ☐ I don't konw
- ☐ I prefer not to answer

What is your current height in **cm**? (please enter numerical value)

____

What is your current weight (without shoes) in **kg**? (please enter numerical value)

____

Intervention Characteristics

The following questions are about your preferences for a health and wellbeing program for women after childbirth. Please answer no matter if you are interested in the program or not.

If there is a health and wellbeing program for women after childbirth, would you be interested in participating?

- ○ Yes
- ○ No

If there is a health and wellbeing program for women after childbirth, how would you like to find out about it? (tick all that apply)

- ☐ Facebook
- ☐ Twitter
- ☐ Instagram
- ☐ WhatsApp
- ☐ WeChat
- ☐ LINE
- ☐ Word of mouth
- ☐ Blog or forum (please specify) ______
- ☐ Newspaper (please specify) ______
- ☐ Playgroup, mother's group or parent's group
- ☐ School, childcare or early learning centre
- ☐ Public library
- ☐ Hospital
- ☐ GP clinic
- ☐ Maternal and child health nurse or centre
- ☐ Others (please specify) ______

When it comes to you personally participating in a health and wellbeing program for women after childbirth, what do you think it would take for you to participate in the program?

(Tick any items on the list that you think apply; you can tick as many or as few as you think appropriate. Some of the items might not look relevant, but that is just because we need to include anything that might possibly apply for some people.)

Capability

I would have to... (tick all that apply)

- [ ] know more about why it was important (e.g. have a better understanding of how foods affect my health)
- [ ] know where to find information
- [ ] know more about how to do it (e.g. have a better understanding of effective ways to increase exercise)
- [ ] know how to create restful time or space for myself
- [ ] have better physical skills (e.g. learn how to cook healthy meals for the family)
- [ ] know how to organise, plan and prioritise (e.g. exercise during child's nap time; incorporate into usual routine such as taking the baby for a walk)
- [ ] have more physical strength (e.g. having the fitness to exercise)
- [ ] have more mental strength (e.g. learn how to resist cravings more)
- [ ] overcome physical limitations (e.g. recovery from childbirth; coping with lack of sleep)
- [ ] overcome mental obstacles (e.g. managing stress or negative thoughts about self)
- [ ] have more physical stamina (e.g. be able to exercise for longer)
- [ ] have more mental stamina (e.g. be able to stick to a plan to eat healthy)
- [ ] something else (please specify) ______

Opportunity

I would have to... (tick all that apply)

- ☐ have more time to do it (e.g. create a specific time during the day to exercise)
- ☐ have a flexible work arrangement (e.g. part-time employment)
- ☐ have enough money to do it (e.g. earn enough to pay for gym membership)
- ☐ have the necessary materials (e.g. exercise equipment)
- ☐ have it more easily accessible (e.g. online access to the intervention)
- ☐ have it incorporated with my baby's appointment (e.g. maternal and child health visits)
- ☐ have a conducive environment to do it (e.g. access to recreational facilities and parks)
- ☐ have more people around me doing it (e.g. be part of a 'crowd' who are doing it)
- ☐ have more triggers to prompt me (e.g. have more reminders to exercise at specific times)
- ☐ have the support of my partner on health issues (e.g. verbal and emotional encouragement)
- ☐ have practical support from others (e.g. help with childcare and chores from partner, family and friends)
- ☐ have someone to hold me accountable
- ☐ something else (please specify) ____________

Motivation

I would have to... (tick all that apply)

- ☐ feel that I want to do it enough (e.g. enjoy eating healthy or exercise)
- ☐ feel that I need to do it enough (e.g. believe that my own health is important; feel the need to prioritise self-care)
- ☐ believe that it would be a good thing to do (e.g. it will help me cope emotionally or make me feel better)
- ☐ believe that it is good for my children (e.g. I am being a good example for my child)
- ☐ develop better plans for doing it (e.g. have clearer and better developed plan for eating healthy)
- ☐ develop a habit of doing it (e.g. get into a pattern of eating healthy without having to think)
- ☐ believe in my ability to do it (e.g. have confidence in my ability to prepare healthy meals)
- ☐ it would have to fit my cultural and/or religious beliefs (e.g. beliefs about the type of food to eat when breastfeeding)
- ☐ something else (please specify) ____________

Who would you like to provide the health and wellbeing program for women after childbirth? (tick all that apply)

- ☐ Someone with expertise in women’s health, e.g. health professional
- ☐ Someone with expertise in children’s health, e.g. health professional
- ☐ Another mum
- ☐ Someone else (please specify) ____________

What information would you like to see included in the health and wellbeing program for women after childbirth? (tick all that apply)

- ☐ Women's health
- ☐ Breastfeeding
- ☐ Caring for my baby
- ☐ Children's health
- ☐ Mum's diet
- ☐ How to lose weight
- ☐ How to prevent weight gain
- ☐ How to maintain weight
- ☐ Preventing diabetes or heart disease
- ☐ Mental health
- ☐ Exercise after birth
- ☐ How to determine the credibility of health information
- ☐ How to set goals and action plans for health
- ☐ How to set aside time for health
- ☐ Self-recording diet or physical activity
- ☐ Monitoring blood tests and other health outcomes
- ☐ Others (please specify) ____________

In addition to the information above, what other aspects would you like to see included in the health and wellbeing program for women after childbirth? (tick all that apply)

- ☐ Someone to monitor my progress
- ☐ Send me reminders and prompts
- ☐ Social support for health
- ☐ Questions to ask my doctor
- ☐ Others (please specify) ____________

When do you think is the best time to start the health and wellbeing program for women after childbirth?

- ○ 6 weeks or earlier
- ○ 7 weeks to 3 months
- ○ 4 to 6 months
- ○ 7 to 12 months
- ○ After 12 months
- ○ Others (please describe) ________

Where would you prefer to receive support on health and wellbeing of mums? (tick all that apply)

- ☐ Online
- ☐ Maternal child health nurse visit
- ☐ Mother's group/playgroup
- ☐ GP clinic
- ☐ Others (please describe) ________

How would you prefer to receive this health and wellbeing support? (tick all that apply)

- ☐ Online information and resource
- ☐ Print information and resource
- ☐ One-on-one video or phone consultation
- ☐ One-on-one face-to-face consultation
- ☐ Group video consultation
- ☐ Group face-to-face consultation
- ☐ Others (please describe) ________

How often would you like to receive this health and wellbeing support?

- Every 6 months
- Every 3 months
- Every month
- Every fortnight
- Every week
- Once off
- Others (please describe)

If this support were to be given through sessions (e.g. a group or individual session), how long should each session last?

- Less than 15 minutes
- Between 15 to 30 minutes
- Between 30 to 45 minutes
- Between 45 to 60 minutes
- More than 60 minutes
- Others (please describe)

If you could access this support over a period of time, how long would you like that period to last?

- <1 month
- 1 month
- 3 months
- 6 months
- 1 year
- Others (please describe)

Wellbeing

These questions concern how you have been feeling over the **past 30 days**. Tick a box across each question that best represents how you have been.

	None of the time	A little of the time	Some of the time	Most of the time	All of the time
About how often did you feel tired out for no good reason?	○	○	○	○	○
About how often did you feel nervous?	○	○	○	○	○
About how often did you feel so nervous that nothing could calm you down?	○	○	○	○	○
About how often did you feel hopeless?	○	○	○	○	○
About how often did you feel restless or fidgety?	○	○	○	○	○
	None of the time	A little of the time	Some of the time	Most of the time	All of the time
About how often did you feel so restless you could not sit still?	○	○	○	○	○
About how often did you feel depressed?	○	○	○	○	○
About how often did you feel that everything was an effort?	○	○	○	○	○
About how often did you feel so sad that nothing could cheer you up?	○	○	○	○	○
About how often did you feel worthless?	○	○	○	○	○

Co-parenting

The following questions relate to how you and your partner work together as parents. Please answer as accurately as possible.

Do you have a partner involved in performing parenting duties?

- ○ Yes
- ○ No

For each item, please select the response that best describes the way you and your partner work together as parents:

	0 = Not true of us	1	2 = A little bit true of us	3	4 = Somewhat true of us	5	6 = Very true of us
I believe my partner is a good parent	○	○	○	○	○	○	○
My relationship with my partner is stronger now than before we had a child	○	○	○	○	○	○	○
My partner asks my opinion on issues related to parenting	○	○	○	○	○	○	○
My partner pays a great deal of attention to our child	○	○	○	○	○	○	○
My partner likes to play with our child and then leave dirty work to me	○	○	○	○	○	○	○

	0 = Not true of us	1	2 = A little bit true of us	3	4 = Somewhat true of us	5	6 = Very true of us
My partner and I have the same goals for our child	○	○	○	○	○	○	○
My partner still wants to do his or her own thing instead of being a responsible parent	○	○	○	○	○	○	○
It is easier and more fun to play with the child alone than it is when my partner is present too	○	○	○	○	○	○	○
My partner and I have different ideas about how to raise our child	○	○	○	○	○	○	○
My partner tells me I am doing a good job or otherwise lets me know I am being a good parent	○	○	○	○	○	○	○

	0 = Not		2 = A little bit		4 =		6 = Very

	[illegible] true of us	1	[illegible] true of us	3	Somewhat true of us	5	[illegible] true of us
My partner and I have different ideas regarding our child’s eating, sleeping, and other routines	○	○	○	○	○	○	○
My partner sometimes makes jokes or sarcastic comments about the way I am as a parent	○	○	○	○	○	○	○
My partner does not trust my abilities as a parent	○	○	○	○	○	○	○
My partner is sensitive to our child's feelings and needs	○	○	○	○	○	○	○
My partner and I have different standards for our child’s behaviour	○	○	○	○	○	○	○

For each item, please select the response that best describes the way you and your partner work together as parents:

	0 = Not true of us	1	2 = A little bit true of us	3	4 = Somewhat true of us	5	6 = Very true of us
My partner tries to show that she or he is better than me at caring for our child	○	○	○	○	○	○	○
I feel close to my partner when I see him or her play with our child	○	○	○	○	○	○	○
My partner has a lot of patience with our child	○	○	○	○	○	○	○
We often discuss the best way to meet our child's needs	○	○	○	○	○	○	○
My partner does not carry his or her fair share of the parenting work	○	○	○	○	○	○	○
	0 = Not true of us	1	2 = A little bit true of us	3	4 = Somewhat true of us	5	6 = Very true of us
When all three of us are together, my partner sometimes competes with me for our child's attention	○	○	○	○	○	○	○
My partner undermines my parenting	○	○	○	○	○	○	○
My partner is willing to make personal sacrifices to help take care of our child	○	○	○	○	○	○	○
We are growing and maturing together through experiences as parents	○	○	○	○	○	○	○
My partner appreciates how hard I work at being a good parent	○	○	○	○	○	○	○
	0 = Not		2 = A little bit		4 =		6 = Very

	[illegible] true of us	1	[illegible] true of us	3	Somewhat true of us	5	[illegible] true of us
When I'm at my wits end as a parent, my partner gives me extra support I need	○	○	○	○	○	○	○
My partner makes me feel like I'm the best possible parent for our child	○	○	○	○	○	○	○
The stress of parenthood has caused my partner and me to grow apart	○	○	○	○	○	○	○
My partner doesn't like to be bothered by our child	○	○	○	○	○	○	○
Parenting has given us a focus for the future	○	○	○	○	○	○	○

These questions ask you to describe things you do when both you and your partner are physically present together with your child (i.e. in the same room, in the car, on outings). Count only times when all three of you are actually within the company of one another (even if this is just a few hours per week).

How often in a typical week, when all 3 of you are together, do you...

	0 = Never	1	2 = Sometimes (once or twice a week)	3	4 = Often (once a day)	5	6 = Very often (several times a day)
Find yourself in a mildly tense or sarcastic interchange with your partner?	○	○	○	○	○	○	○
Argue with your partner about your child, in the child's presence?	○	○	○	○	○	○	○
Argue about your relationship or marital issues unrelated to your child, in the child's presence?	○	○	○	○	○	○	○
One or both of you say cruel or hurtful things to each other in front of the child?	○	○	○	○	○	○	○
Yell at each other within earshot of the child?	○	○	○	○	○	○	○

These questions are about how satisfied you are with the way you and your partner divide or share the family tasks. Please rate your satisfaction below.

	0 = Very dissatisfied	1 = Moderately dissatisfied	2 = Slightly dissatisfied	3 = Neutral	4 = Slightly satisfied	5 = Moderatel satisfied
In general, how satisfied are you with the way you and your partner divide the family tasks?	○	○	○	○	○	○
How satisfied are you with the way that you and your partner talk about and listen to each other when it comes to sharing family tasks and responsibilities, regardless of how the tasks are actually divided?	○	○	○	○	○	○

Physical activity and sedentary time

Please state **how many times** you did each type of activity **last week**. Only count **activities that lasted for 10 minutes or more**.

*(For example: If you go for a brisk walk twice a week, please record '2' under 'number of times'; If you did **not** do an activity, please write '0' in the boxes.)*

	Number of times
**Walking briskly** (for recreation or exercise, or to get from place to place)	
**Moderate leisure activity** (like social tennis, moderate exercise classes, recreational swimming, dancing)	
**Vigorous leisure activity** (that makes you breathe harder or puff and pant like aerobics, competitive sport, vigorous cycling, running, swimming)	
**Vigorous household or garden chores** (that make you breathe harder or puff and pant)	

Please state **the total time** you spent doing each type of activity **last week**. Only count **activities that lasted for 10 minutes or more**; add up all the times you spent in each activity to get the total time for each activity.

*(For example: If you went for a brisk walk for 1 hour in total last week, please record '1' under 'hours' and '0' under 'minutes'; If you did **not** do an activity, please write '0' in the boxes.)*

	Hours	Minutes
**Walking briskly** (for recreation or exercise, or to get from place to place)		
**Moderate leisure activity** (like social tennis, moderate exercise classes, recreational swimming, dancing)		
**Vigorous leisure activity** (that makes you breathe harder or puff and pant like aerobics, competitive sport, vigorous cycling, running, swimming)		
**Vigorous household or garden chores** (that make you breathe harder or puff and pant)		

Please think about **how much time in total** you spend **sitting** during **each day** while at home, at work, while getting from place to place or during your spare time (like visiting friends, driving, reading, watching television, or working at a desk or computer).

(For example: If you spend 6 hours and 20 minutes sitting on a usual weekday, please record '6' under 'hours' and '20' under 'minutes'.)

	Hours	Minutes
On a usual week day		
On a usual weekend day		

Dietary Intake

Please record the estimated number of serves you eat from each food group. **Account for all food eaten, including the ingredients added to recipes, eaten in mixed meals and in restaurant or take away meals.** You can provide an estimate of the serves you eat in **1 day OR 1 week**. If you do **not** eat a certain group of food, please write '0' in the box.

Grains: bread, cereal, rice, pasta etc

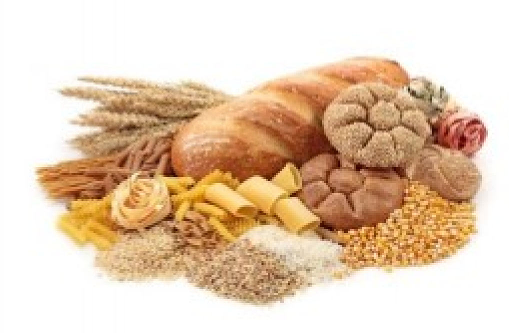

Examples of 1 serve

1 slice (40g)	bread
1/2 medium (40g)	bread roll or flat bread
1/2 cup (120g)	cooked porridge
2/3 cup (30g)	wheat breakfast cereal flakes
1/4 cup (30g)	muesli
1 (60g)	crumpet
1/2 cup (75-120g)	cooked rice, pasta, noodles, quinoa, barley or other grains
3-6 (35g)	crisp breads (3 Ryvita or 6 Vitawheats/Cruskits)

Estimated number of serves (fill in **one option only**)

per day

per week

Vegetable and legumes

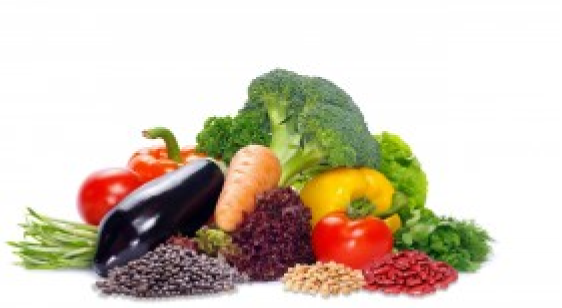

Examples of 1 serve

1/2 cup	cooked green or orange vegetables (e.g. broccoli, spinach, carrot, pumpkin etc.)
1 cup	raw green leafy vegetables or salad vegetables
1/2 cup	cooked dried or canned beans, peas or lentils
1 small or 1/2 medium	potato/starchy vegetable (e.g. sweet potato, sweet corn, taro or cassava)
1 medium	tomato

Estimated number of serves (fill in **one option only**)

per day

per week

Fruit

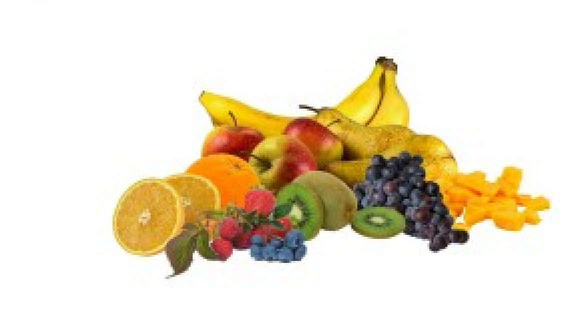

Examples of 1 serve

1 medium	apple, banana, orange or pear
2 small	apricots, kiwi fruit or plum
1 cup	berries, diced fruit pieces or canned fruit
30g	dried fruit (e.g. 4 dried apricot halves, 1.5 tablespoons sultanas)
1/2 cup (125ml)	fruit juice (not made from fruit concentrate)

Estimated number of serves (fill in **one option only**)

per day

per week

Milk, yoghurt, cheese or alternatives

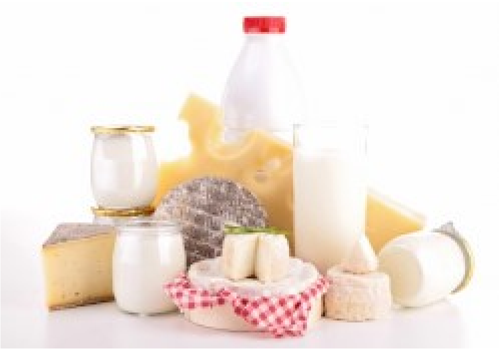

Examples of 1 serve

1 cup (250mL)	dairy milk or calcium fortified dairy alternative milk (soy, rice, almond etc.) or other cereal drink
2 slices cheese or 4 cubes (3x2cm) (40g)	hard cheese (e.g. cheddar, gouda)
1/2 cup (120g)	ricotta cheese
3/4 cup (200g; roughly 1 small tub)	dairy yoghurt, or calcium fortified dairy alternative yoghurt
**OR non-dairy alternatives:**	
1/2 cup (100g)	almonds with skin
1/3 cup (75-80g)	canned pink or Australian salmon with bones
100g	firm tofu

Estimated number of serves (fill in **one option only**)

per day

per week

Meat, fish, poultry, eggs, nuts and legumes

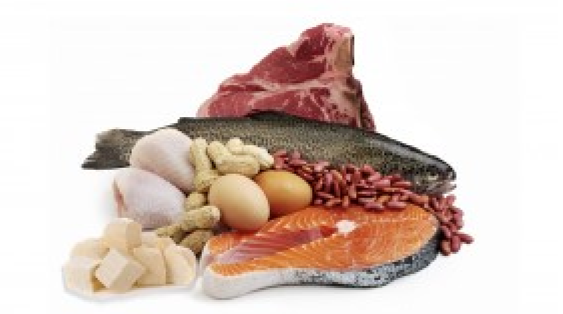

Examples of 1 serve

65g	cooked red meat such as beef, veal, lamb, pork, kangaroo or goat (approx. 90-100g raw) (e.g. 1/2 cup lean mince, 2 small

	chops or 2 slices roast meat)
80g	cooked poultry such as chicken or turkey (approx. 100g raw weight) (e.g. 1/2 medium chicken breast)
100g	cooked fish fillet (approx. 115g raw weight) or 1 small can of fish
2 large (120g)	eggs
1 cup (150g)	cooked or canned beans, peas, lentils (e.g. chickpeas or split peas)
170g	tofu
30g	nuts, seeds or nut butters/seed paste (e.g. peanut butter, tahini)

Estimated number of serves (fill in **one option only**)

per day

per week

Extras/Discretionary choices

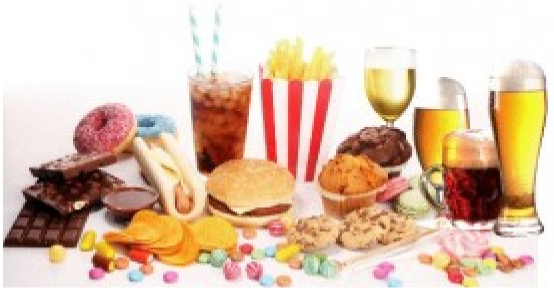

Examples of 1 serve

1 tablespoon	butter, margarine, jam or honey
2 scoops (75g)	regular ice cream
2 slices	processed meat (e.g. salami, ham etc)
2 thin	sausages

1/3 (60g)	individual meat pie or pasty
12	hot chips
1 small	slice of plain cake or doughnut
2-3 small	sweet biscuits
5-6	lollies
1 small (25g)	bar of chocolate
**Drinks:**	
1 can (375mL)	soft drink (excluding diet or sugar free options)
200ml (1 large glass)	wine
400ml (approx. 1 bottle)	standard beer
60ml (2 shots)	spirits

Estimated number of serves (fill in **one option only**)

per day ______

per week ______

Sleep

The following questions are about your sleep quality. Tick one box across each questions that most accurately describes your sleep quality **in the past 7 days**.

In the past 7 days,

	Very poor	Poor	Fair	Good	Very good
My sleep quality was...	○	○	○	○	○

In the past 7 days,

	Not at all	A little bit	Somewhat	Quite a bit	Very much
My sleep was refreshing	○	○	○	○	○
I had a problem with my sleep	○	○	○	○	○
I had difficult falling asleep	○	○	○	○	○
I had a hard time getting things done because I was sleepy	○	○	○	○	○
I had problems during the day because of poor sleep	○	○	○	○	○
I had a hard time concentrating because of poor sleep	○	○	○	○	○
I was sleepy during the daytime	○	○	○	○	○

Risk perception for cardiovascular disease and type 2 diabetes

The following questions are about your opinion on your risk of diabetes. Tick the box that most accurately describes your opinion on your risk.

What do you think your risk or chance is for getting diabetes over the next 10 years?

- ○ Almost no risk
- ○ Slight risk
- ○ Moderate risk
- ○ High risk

If you don’t change your lifestyle behaviours, such as diet or exercise, what is your risk or chance of getting diabetes over the next 10 years?

- Almost no risk
- Slight risk
- Moderate risk
- High risk

Have you recently made changes in any lifestyle behaviours that you believe will lower your chance of getting diabetes?

- Yes
- No

Are you planning to make changes in any lifestyle behaviours in the near future that you believe will lower your chances of getting diabetes?

- Yes
- No

The following questions are about your opinion on your risk of cardiovascular diseases (e.g. heart disease, hypertension, stroke). Tick the box that most accurately describes your opinion on your risk.

What do you think your risk or chance is for getting cardiovascular diseases (e.g. heart disease, hypertension, stroke) over the next 10 years?

- Almost no risk
- Slight risk
- Moderate risk
- High risk

If you don’t change your lifestyle behaviours, such as diet or exercise, what is your risk or chance of getting cardiovascular diseases (e.g. heart disease, hypertension, stroke) over the next 10 years?

- Almost no risk
- Slight risk
- Moderate risk
- High risk

Have you recently made changes in any lifestyle behaviours that you believe will lower your chance of getting cardiovascular diseases (e.g. heart disease, hypertension, stroke)?

- Yes
- No

Are you planning to make changes in any lifestyle behaviours in the near future that you believe will lower your chances of getting cardiovascular diseases (e.g. heart disease, hypertension, stroke)?

- Yes
- No

Powered by Qualtrics
