# Supplementary material for: Ethnic Differences in Facilitators and Barriers to Lifestyle Management After Childbirth: A Multi-Methods Study Using the TDF and COM-B Model
Source: Nutrients. 2025 Jan 14;17(2):286. doi: 10.3390/nu17020286 (PMC11769254; doi:10.3390/nu17020286)
Supplement: Supplementary file 1 [file nutrients-17-00286-s001.zip › Table S1.pdf]

Table S1. Demographic characteristics of Indigenous participants in the survey (n=27)

Characteristics	Mean±SD or n (%)
Age (years)	29.4±5.9
Postpartum age (years)	2.0±1.6
Number of children living in the household	
1	4 (14.8)
2	12 (44.4)
≥ 3	11 (40.7)
Born in Australia	
No	3 (11.1)
Years lived in Australia	
≤ 5 years	0 (0.0)
6 to 10 years	1 (3.7)
≥ 11 years	26 (96.3)
Marital status	
Never married	5 (18.5)
Married	8 (29.6)
De facto	11 (40.7)
Separated or divorced	3 (11.1)
Education	
High school	18 (66.7)
Diploma	3 (11.1)
Bachelor	3 (11.1)
Postgraduate	3 (11.1)
Employment	
Full time	7 (25.9)
Part time	9 (33.3)
Unemployed	11 (40.7)
Household income per year ($)	
≤ 49,999	10 (37.0)
50,000 to 99,999	10 (37.0)
≥ 100,000	7 (25.9)
Prefer not to answer	0 (0.0)

SD, standard deviation.

Indigenous participants included Australian Aboriginal, Torres Strait Islanders, Maori and Pacific Islanders.
