# Supplementary material for: Ethnic Differences in Facilitators and Barriers to Lifestyle Management After Childbirth: A Multi-Methods Study Using the TDF and COM-B Model
Source: Nutrients. 2025 Jan 14;17(2):286. doi: 10.3390/nu17020286 (PMC11769254; doi:10.3390/nu17020286)
Supplement: Supplementary file 1 [file nutrients-17-00286-s001.zip › Table S2.pdf]

Table S2. Survey responses on capability, opportunity and motivation for participation in lifestyle management after childbirth according to the COM-B and TDF domains in Indigenous participants (n=27)

COM-B constructs	TDF domains	Questionnaire statement: *I would have to…*	n (%)
Psychological capability	Knowledge	know more about why it was important	12 (44.4)
		know more about how to do it	15 (55.6)
	Skills	know where to find information	18 (66.7)
		know how to create restful time or space for myself	17 (63.0)
		have better physical skills	15 (55.6)
	Behavioural regulation	know how to organise, plan and prioritise	20 (74.1)
		have more mental strength	21 (77.8)
		have more mental stamina	17 (63.0)
Physical capability	Skills	have more physical strength	17 (63.0)
		have more physical stamina	14 (51.9)
		overcome physical limitations	15 (55.6)
		overcome mental obstacles	17 (63.0)
Physical opportunity	Environmental context and resources	have more time to do it	20 (74.1)
		have a flexible work arrangement	10 (37.0)
		have enough money to do it	18 (66.7)
		have the necessary materials	13 (48.2)
		have it more easily accessible	9 (33.3)
		have it incorporated with my baby's appointment	5 (18.5)
		have a conducive environment to do it	7 (25.9)
Social opportunity	Social influences	have more people around me doing it	9 (33.3)
		have more triggers to prompt me	12 (44.4)
		have the support of my partner on health issues	15 (55.6)
		have practical support from others	13 (48.2)
		have someone to hold me accountable	8 (29.6)
Reflective motivation	Intentions	feel that I need to do it enough	17 (63.0)
	Beliefs about consequences	believe that it would be a good thing to do	18 (66.7)
		believe that it is good for my children	17 (63.0)
	Beliefs about capabilities	believe in my ability to do it	17 (63.0)
	Goals	develop better plans for doing it	16 (59.3)
	Social/professional role and identity	it would have to fit my cultural and/or religious beliefs	7 (25.9)
Automatic motivation	Emotion	feel that I want to do it enough	19 (70.4)
		develop a habit of doing it	17 (63.0)

COM-B, Capability Opportunity Motivation – Behaviour; TDF, Theoretical Domains Framework.
Indigenous participants included Australian Aboriginal, Torres Strait Islanders, Maori and Pacific Islanders.
