# Supplementary material for: Ethnic Differences in Facilitators and Barriers to Lifestyle Management After Childbirth: A Multi-Methods Study Using the TDF and COM-B Model
Source: Nutrients. 2025 Jan 14;17(2):286. doi: 10.3390/nu17020286 (PMC11769254; doi:10.3390/nu17020286)
Supplement: Supplementary file 1 [file nutrients-17-00286-s001.zip › Table S3.pdf]

Table S3. Location of residence and ethnicity of survey and interview participants

	Survey (n=478)	Interview (n=17)
**State or territory**		
Victoria	134 (28.0)	15 (88.2)
New South Wales	142 (29.7)	0 (0.0)
Queensland	96 (20.1)	2 (11.8)
South Australia	29 (6.1)	0 (0.0)
Western Australia	51 (10.7)	0 (0.0)
Australian Capital Territory	10 (2.1)	0 (0.0)
Tasmania	11 (2.3)	0 (0.0)
Northern Territory	5 (1.1)	0 (0.0)
**Ethnicity**		
Oceanian		
Australian	229 (47.9)	7 (41.2)
New Zealander	10 (2.1)	1 (5.9)
Asian		
North-East Asian	27 (5.7)	3 (17.6)
South-East Asian	66 (13.8)	1 (5.9)
Southern and Central Asian	81 (17.0)	5 (29.4)
Other		
North-West European	11 (2.3)	NA
Southern and Eastern European	7 (1.5)	NA
North African and Middle Eastern	9 (1.9)	NA
North American	9 (1.9)	NA
South American	12 (2.5)	NA
Central American	1 (0.2)	NA
Caribbean Islander	1 (0.2)	NA
Central and West African	1 (0.2)	NA
Southern and East African	10 (2.1)	NA
Mixed	4 (0.8)	NA

NA, not applicable. Data are presented as n (%).
