# Supplementary material for: Ethnic Differences in Facilitators and Barriers to Lifestyle Management After Childbirth: A Multi-Methods Study Using the TDF and COM-B Model
Source: Nutrients. 2025 Jan 14;17(2):286. doi: 10.3390/nu17020286 (PMC11769254; doi:10.3390/nu17020286)
Supplement: Supplementary file 1 [file nutrients-17-00286-s001.zip › Table S4.pdf]

Table S4. Codes, subthemes and themes on women's perspectives derived from interviews

COM-B constructs	TDF domains	Initial codes	Subthemes	Themes
Psychological capability	Knowledge, skills	Facilitator: Knowing health consequences	Facilitator: Knowledge of healthy lifestyle	Having and obtaining knowledge of healthy lifestyle
		Facilitator: Knowing benefits of healthy lifestyle		
		Barrier: Not knowing where to find information	Barrier: Lack of skills to find information	
	Behavioural regulation	Facilitator: Prioritising, organising or planning	Facilitator: Ability to prioritise, organise and plan	Prioritising, organising and planning for healthy lifestyle
		Facilitator: Regularity, consistency and routine	Facilitator: Incorporate into routine life	
		Facilitator: Fitting it in where you can		
		Facilitator: Walking the dog		
Physical capability	Skills	Barrier: Lack of sleep	Barrier/Facilitator: Fatigue and sleep issues	Fatigue, lack of sleep and mental health challenges
		Facilitator: Enough or good sleep		
		Barrier: Tiredness		
		Barrier: Stress and mental burden	Barrier/Facilitator: Mental health issues	
		Facilitator: Addressing mental health		
Physical opportunity	Environmental context and resources	Barrier: Busy and time	Barrier/Facilitator: Time availability	Limited time availability with competing priorities
		Facilitator: Time on own to have right headspace		
		Barrier: Motherly duties and childcare needs	Barrier: Childcare needs	
		Barrier: Nature of work	Barrier/Facilitator: Work arrangement	
		Facilitator: Flexible work		
		Facilitator: Good work-life balance		
		Facilitator: Working from home during COVID		
		Barrier: Where they live – poor exercise facilities	Barrier/Facilitator: Practical resources in living environment	Physical access to healthy lifestyle resources
		Facilitator: Where they live – infrastructure to support exercise and wellbeing		
		Facilitator: Food delivered to home		

		Facilitator: Exercising at home, online or phone app exercise program		
		Barrier: Unsafe environment for walking	Barrier: Unsafe living environment	
		Barrier: Poor weather conditions	Barrier: Poor weather conditions	
		Barrier: COVID and lockdown – reduced opportunity to exercise	Barrier: Reduced exercise due to COVID lockdown	
		Barrier: Financial costs	Barrier/Facilitator: Financial influences	
		Facilitator: Adequate finances		
Social opportunity	Social influences	Facilitator: Babysitting and supportive childcare service	Facilitator: Childcare service support	Practical support on childcare and household chores
		Barrier: Busy partner	Barrier/Facilitator: Partner support on childcare and household chores	
		Facilitator: Supportive partner coparenting		
		Barrier: Lack of family support	Barrier/Facilitator: Family support on childcare	
		Facilitator: Practical family support		
		Facilitator: Health service support	Facilitator: Health professional support	Mental and wellbeing support especially for migrants
		Facilitator: Partner sharing same health values	Facilitator: Partner support on health issues	
		Facilitator: Emotional support from family	Facilitator: Emotional support from family, friends and church	
		Facilitator: Peer support		
		Facilitator: Spiritual and church support		
		Facilitator: Having accountability from others	Facilitator: Accountability on health	
		Barrier: Lack of social connection especially as a migrant	Barrier: Lack of social support especially for migrants	
		Barrier: COVID and lockdown – lack of social interaction		
		Barrier: Social expected roles of mother	Barrier: Parenting roles in Asian cultures	Social norms around parenting and postpartum practices in
		Barrier: Postpartum practices	Barrier: Traditional postpartum practices in	

			Asian cultures	Asian cultures
Reflective motivation	Intentions, goals	Barrier: Difficulty prioritising self	Barrier: Difficulty prioritising self	Difficulties with prioritising self and maintaining motivation
		Barrier: Lack of motivation	Barrier/Facilitator: Maintaining motivation	
		Barrier: Lack of sustainability		
		Facilitator: Self-talk self-motivation		
		Facilitator: Having a goal		
Automatic motivation	Emotion	Facilitator: Exercise as emotional coping strategy	Barrier/Facilitator: Enjoyment in exercise, cooking or eating	Enjoyment in exercise or eating behaviours
		Facilitator: Finding exercise that appeals		
		Facilitator: Enjoy cooking		
		Barrier: Snacking, sweet craving, taste preference		
